# Where Arsenic May Be Found after Death

**Published:** 1855-12

**Authors:** 


Where Arsenic may be found after Death.—With regard to the customary limitation of the search for arsenic, in cases of suspected poisoning, to the stomach and its contents, Buchner is of opinion that it is insufficient to justify an opinion when negative results are obtained. In one instance he found that arsenic was present in the stomach and duodenum only in such amount as to be barely recognized; still, when different parts of the intestinal canal were examined, arsenic was found without difficulty. The lower portion of the intestine contained a tolerable quantity of slimy substance, colored yellow by bile, and here the arsenic was found in largest amount; while in the upper portion of the intestine, which was empty, like the stomach, the amount of arsenic was smaller. The author considers it not improbable that in this instance arsenic had been absorbed and excreted by the liver into the intestine.—*Pharmaceutical Journal.*

